# The effects of gaze stability exercises on balance, gait ability, and fall efficacy in patients with chronic stroke: A 2-week follow-up from a randomized controlled trial

**DOI:** 10.1097/MD.0000000000039221

**Published:** 2024-08-09

**Authors:** Zhe Cui, Ying-Ying Tang, Myoung-Ho Lee, Myoung-Kwon Kim

**Affiliations:** a Department of Rehabilitation Sciences, Graduate School, Daegu University, Jillyang, Gyeongsan, Gyeongbuk, Republic of Korea; b Department of Physical Therapy, College of Rehabilitation Sciences, Daegu University, Jillyang, Gyeongsan, Gyeongbuk, Republic of Korea.

**Keywords:** balance exercise, gait, gaze stability exercise, stroke

## Abstract

**Background:**

This study aimed to examine the effect of gaze stability exercises on balance, gait ability, and fall efficacy in patients with chronic stroke, as well as to investigate whether any observed effects were maintained 2 weeks later.

**Methods:**

In this experiment, 30 chronic stroke patients were selected. The patients were randomly divided into 3 groups (10 patients in each group). All patients in the 3 groups performed basic neurodevelopmental treatment. Group 1 performed balance exercises accompanied by gaze stability exercises. Group 2 performed gaze stability exercises, and group 3 performed balance exercises. Each exercise program for 40 minutes 3 times a week for 4 weeks. After the intervention period, the patient’s balance, gait ability, and fall efficacy were measured again. In order to know whether the training effect is maintained, a 2-week follow-up test was conducted after the training.

**Results:**

The results of this study showed that there was a significant improve in balance (overall stability index, limit of stability test, and Berg Balance Scale), gait ability (gait velocity, cadence, step time and step length, Timed Up and Go [TUG] test), and fall efficacy over the different time within the 3 groups. The effect was observed to be maintained in follow-up tests after 2 weeks. In the comparison among 3 groups, the overall stability index, limit of stability test in the balance test and the gait velocity, cadence, step time, step length and Timed Up and Go test in the gait test all showed statistically significant differences, and the other items did not have significant differences. In most of the assessments, group 1 that used balance exercise combined with gaze stability exercise showed a better improvement than the other 2 groups.

**Conclusion:**

As a result, for stroke patients, gaze stability exercise is an effective arbitration method to improve balance and gait ability and fall efficacy. With balance exercise combined with gaze stability exercise, a greater effect can be seen than with gaze stability exercise or balance exercise alone. Thus, this combination exercise program can be recommended as effective.

## 1. Introduction

### 1.1. Necessity of study

Stroke refers to the blockage or bleeding of a blood vessel in the brain. When blood flow to the brain is obstructed, it results in a lack of oxygen, leading to the death of brain cells. This cellular death causes loss of brain function, which can result in impairment of memory and muscular control.^[1]^ Stroke is associated with significant neurological deficits, such as sudden weakness or numbness in the face, arm, or leg, typically affecting one side of the body. Other factors include confusion, severe headache accompanied by unexplained fainting, difficulty speaking and walking, and loss of coordination and balance. All of these symptoms disturb the patient’s recovery.^[2,3]^ Patients with hemiplegia, due to the movement obstacle of the paralyzed side, experience reduced adjustment and coordination abilities of the upper and lower limbs, making it challenging for them to respond quickly when their posture is disturbed.^[4]^ Postural imbalance and gait disturbances after a stroke are significant risk factors for falls. The high incidence of falls in these patients is well-documented in the literature and has notable social and economic impacts.^[5,6]^ When falls occur, individuals experience increased fear of falling, loss of self-confidence, and activity limitations, leading to greater dependence on others in daily life and, consequently, a reduced quality of life.^[7]^

Balance is a highly complex function that involves both the musculoskeletal and nervous systems. Functional factors include vision, hearing, vestibular sensation, proprioception, visuospatial perception, environmental changes, muscle strength, endurance, joint flexibility, and the central nervous system’s response to precise stimuli.

Balance involves the integration of 3 major systems. Firstly, sensory information that evaluates the body’s position and movement in space is processed by the sensory system (vision, somatosensory, vestibular). Secondly, the ability to produce forces to control body position is derived from the musculoskeletal system (strength, range of motion, flexibility, endurance). Lastly, the central processing system decides on effective and timely responses.^[8]^ Specifically, as components of the body’s sensory system, visual, vestibular, and somatosensory inputs play important roles in postural stability.^[9]^

Balance exercises are among the four types of recommended exercises, alongside strength, aerobic, and flexibility exercises, aimed at enhancing overall health and physical capabilities.^[10]^ Balance training is conducted to reduce the risk of falls and injuries. These programs focus on enhancing balance control during daily tasks, thereby improving self-confidence in preventing falls, reducing fear of falling, and increasing walking speed. They also aim to enhance physical function and overall quality of life.^[11]^ Previous reviews have shown the positive effect of balance training on balance performance, gait, and function of individuals post-stroke.^[11–13]^ Kong et al^[14]^ reported that balance training on different support surface led to greater improvement in balance and walking ability than each of the other methods in the patients with chronic stroke. Halvarsson et al^[11]^ reported that balance training is considered an important component of fall prevention programs. Their study showed that it effectively improves different aspects of postural control and leads to a significant reduction in fall rates among the elderly.

In addition, intervention methods are emerging to improve trunk stability and balance in stroke patients associated with visual movement.^[1]^ Vision has an important role in controlling spatial orientation and balance. Using vision to monitor body position in relation to the surrounding environment enables individuals to detect and respond to subtle shifts in body posture. The major elements of balance include not only the sense of sight but also the vestibular organ and somatosensory.^[15]^ The vestibular system aids in maintaining postural stability and visual stabilization through the vestibulo-spinal reflex (VSR) and the vestibulo-ocular reflex (VOR), respectively. The VOR is the primary mechanism for gaze stability.^[2]^ The VSR initiates the contraction of anti-gravity muscles to support postural stability, while the VOR generates eye movements to preserve clear visual acuity during head movements. Additionally, the cervico-ocular reflex interacts with the vestibular nuclei and can also induce eye movements.^[16]^ Gaerlan et al indicated that the visual components of these 3 systems are crucial for maintaining balance in a standing position.^[17]^ However, stroke patients often experience impairments in various oculogyrations and have difficulties with the pendulum movement of the eyes. These issues are considered obstacles to improving functional ability and movement recovery.^[18]^ Gaze stability is essential for coordinating movements of the head, trunk, and pelvis during walking. Individuals post-stroke have been observed to display abnormal coordination of axial segments and pelvic rotations during head movements, potentially contributing to balance changes during gait. Reduced stability of the trunk and head post-stroke also impairs the quality of visual information, which can further compromise balance.^[2]^ Therefore, eye movements can be used as an arbiter to improve this condition.^[19]^ Eye movements impact balance depending on the type of movement performed. Humans have 3 visuomotor options (visual fixation, smooth pursuit eye movements, and saccades), and previous studies have shown that these eye movements can affect not only body balance but also various functions associated with different types of eye movements.^[20]^

Recently, the benefits of gaze stability exercises have gained recognition for enhancing balance in patients undergoing vestibular rehabilitation for vestibular dysfunction.^[21]^ It has been established that eye movement exercises help stabilize the eye position relative to the head during postural control or locomotion,^[22]^ leading to improved balance ability, confidence, and enhanced cognitive function.^[21]^ One method that can potentially initiate sensory reweighting is the gaze stabilization exercise. Gaze stability exercises are not only for improving postural stability and gaze stability during head movement in healthy young adults but also for enhancing balance function in the static and dynamic movements of healthy elderly individuals. They have been shown to reduce the perceived disability in individuals with unilateral vestibular dysfunction.^[2,23]^

While the significance of eye movements for postural control has been highlighted, previous studies have primarily focused on healthy adults. There is limited research on the impact of eye movements on postural stability in chronic stroke patients. Therefore, this study examined the effects of gaze stability exercises on balance, gait ability, and fall efficacy in patients with chronic stroke, and observed whether the effects were maintained 2 weeks later.

### 1.2. Purpose of study

This study aimed to examine the effect of gaze stability exercises on balance, gait ability, and fall efficacy in patients with chronic strokeand observed whether the effects were maintained 2 weeks later.

## 2. Methods

### 2.1. Experimental subjects

Based on the data from the pilot study, the necessary sample size was determined using G*Power software. Assuming a 0.05 α-error probability with a statistical power of 0.80 and an effect size of 0.61, a sample size of 30 chronic stroke patients was found to be required for the study. The patients were randomly divided into 3 groups (10 patients in each group). All patients in the 3 groups performed basic neurodevelopmental treatment. Group 1 performed balance exercises accompanied by gaze stability exercises. Group 2 performed gaze stability exercises and group 3 performed balance exercises. Prior to participation, all participants reviewed and signed consent forms approved by the Institutional Review Board of Daegu University (IRB No.1040621-202307-HR-050).

The selection criteria were as follows: Patients who first had been diagnosed as a stroke and passed for more than 6 months; patients without visual, hearing, and vestibular disorders; patients without cognitive impairment and more than 24 points in the Korean Version of Mini Status Examination; ability to stand and walk 10 m independently.^[1,24]^

The exclusion criteria were as follows: Patients with hemi-neglect and half blindness; patients with somatosensory defects that affect balance ability; and patients with a degenerative disease and musculoskeletal system impairment affecting the standing balance.^[1]^

### 2.2. Experimental procedure

#### 2.2.1. Experimental design

**Figure 1.**
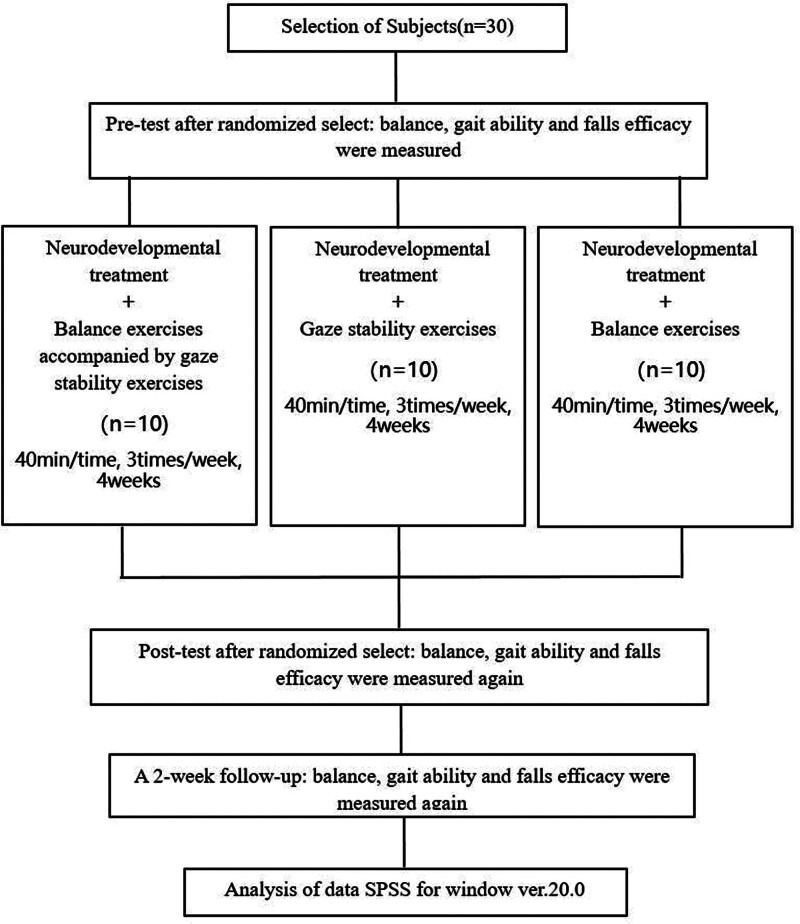
Study flowchart.

In this experiment, 30 chronic stroke patients were selected. The patients were randomly divided into 3 groups (10 patients in each group). The 3 exercise programs were written on slips of paper, and the patients were assigned to each program by drawing lots. Before the intervention period, the patient’s balance, gait ability, and fall efficacy were measured. All patients in the 3 groups performed basic neurodevelopmental treatment for 30 minutes. Group 1 performed balance exercises accompanied by gaze stability exercises for 10 minutes. Group 2 performed gaze stability exercises for 10 minutes and group 3 performed balance exercises for 10 minutes. Each exercise program for 40 minutes 3 times a week for 4 weeks. After the intervention period, the patient’s balance, gait ability, and fall efficacy were measured again. In order to know whether the training effect is maintained, a 2-week follow-up test was conducted after the training (Fig. 1).

#### 2.2.2. Gaze stability exercise

Description of the gaze stability exercises based on previous papers.^[2]^ The exercise program consists of 8 different gaze stability exercises (Table 1). Participants should perform the exercises in standing position, each exercise should be repeated 10 times. The exercise program for 10 minutes, 30-second rest intervals were allowed between the exercises, 3 times a week for 4 weeks. Participants are instructed to maintain focus on the target during exercise and to slowly move the target or head. If participants experience any type of imbalance or dizziness, pause briefly during the exercise and resume if possible. All exercises are preceded by instructions on correct execution of movements. If needed, a third party assists participants in performing exercises accurately and safely.

**Table 1:** Gaze stability exercise

Exercises	Procedures
Exercise 1	Moving the eyes horizontally between 2 stationary targets while keeping the head still—saccadic eye movement exercises.
Exercise 2	Moving the eyes vertically between 2 stationary targets while keeping the headstill—saccadic eye movement exercises.
Exercise 3	Moving the target horizontally and tracking it with the eyes while keeping the head still—smooth pursuit exercises.
Exercise 4	Moving the target vertically and tracking it with the eyes while keeping the head still—smooth pursuit exercises.
Exercise 5	Moving the head horizontally while keeping the look on a stationary target—adaptation exercises.
Exercise 6	Moving the head vertically while keeping the look on a stationary target—adaptation exercises.
Exercise 7	Moving the head and target in opposite directions horizontally while tracking the target with the eyes—adaptation exercises.
Exercise 8	Moving the head and target in opposite directions vertically while tracking the target with the eyes—adaptation exercises.

#### 2.2.3. Balance exercise

Balance exercises were taken from previous studies.^[13,25]^ The balance exercises were focused on trunk stabilization and weight transfer to the paretic leg and consisted of 2 exercises (Table 2). The exercise program for 10 minutes, 30-second rest intervals were allowed between the exercises, 3 times a week for 4 weeks.

**Table 2:** Balance exercise

Exercises	Procedures
Exercise 1	Patient asked to stand upright on Airex balance pad to maintain posture.
Exercise 2	Standing on one leg and raising other leg to a stair; alternate both lower limbs.

#### 2.2.4. Neurodevelopment treatment

All groups considered the level of individual function implementation of patients with chronic stroke and received routine neurodevelopmental physiotherapy for 30 minutes 3 times a week for 4 weeks. Neurodevelopmental physiotherapy program included upper extremity activities, trunk exercises, sitting, standing, and walking exercises, balance exercises, and lower extremity exercises tailored to the patient’s functional requirements. Strengthening exercises for weakened areas for postural control training and functional movement. And training for the improvement of the optimal activities and functions of daily living according to the characteristics of the individual. The exercise, conducted at the individual level by a physical therapist with more than 5 years of service at the hospital,^[1]^ was carried out with a focus on safety.

### 2.3. Experimental instrumentation

#### 2.3.1. Biodex Balance System

The Biodex Balance System SD (Biodex Medical System Inc., Shirley) is a device that allows balance training and evaluation while standing on a circular platform. The postural stability test is used to check the patient’s ability to adjust postures to minimize postural fluctuations from the center of the circular platform to all directions, and is expressed as a stability index. Stability index refers to the ability to control posture in all directions. The higher the stability index, the greater the posture fluctuation. Overall stability index represents the patient’s ability to maintain balance in all directions. The intratester reliability of the overall stability index was *r* = 0.84.^[4]^

The limit of stability test is the process of the central target point moving toward 8 randomly flashing signal points, keeping the movement as straight as possible and returning to the center target point. The balance ability was evaluated with the overall score among the limit of stability variables because the effect of low and high scores on the average was small and the measurement error was very small. Each direction has a maximum score of 100. A higher score on the limit of stability test indicates greater balance ability. The sensitivity and specificity of the limit of stability test were 50% and 88.5%, respectively.^[26]^

The evaluation method is to have the subject stand on a circular platform, which has test levels ranging from 1 to 8. Level 1 represents the most frequent movement, while level 8 signifies the least movement. This study, targeting patients with central nervous system injuries, conducted the evaluation at level 8, which features minimal movement and the lowest risk factor. And then put the heel position on the left D6 and the right D16, with the angle of 2 feet being 20°. Subjects were instructed to gaze at a black dot displayed on the LCD screen for 20 seconds and to maintain posture to minimize postural fluctuations. The postural stability test was conducted with eyes open and closed, and the limit of stability test was conducted with eyes open. After 3 times of measurements, the stability index is calculated with the average value.^[4,27]^

#### 2.3.2. Timed Up and Go (TUG) test

TUG test is used for dynamic balance measurement. Previous studies indicated, respectively, the test–retest reliability of the standing up and walking test in stroke patients was reported to be 0.95, and reported that the test–retest reliability of the TUG test was excellent (intraclass correlation coefficient = 0.99^[28]^). The participants were instructed to stand up from a chair with armrests, walk 3 m, turn around, return to the chair, and sit down at a fast and safe speed. The total time required to complete the task was then calculated. Each subject completed the TUG test 3 times and calculated the average time value. To avoid fatigue allow 1-minute rest per test.^[29]^

#### 2.3.3. Berg Balance Scale (BBS)

The BBS is a widely used scale for evaluating balance-related disorders. It consists of a 14-item balance scale with a 5-point grading (0–4) for each item. A score of 0 indicates the need for maximal assistance or safety concerns in task completion, while a score of 4 indicates independent and safe task performance. The maximum score is 56 points, with higher scores reflecting better performance. The BBS has demonstrated excellent validity and outstanding intra-rater and inter-rater reliability (interclass correlation = 0.99). It is extensively utilized as a tool to measure balance function.^[30]^

#### 2.3.4. Gait measurement

The GAITRite system (CIR Systems Inc., Havertown) automates measuring temporal and spatial gait parameters via an electronic walkway connected to the USB port of a Windows computer. The standard GAITRite electronic walkway contains 8 sensor pads encapsulated in a rolled-up carpet to produce an active area 61 cm wide and 488 cm long. As the subject ambulated across the walkway, the sensors continuously captured each footfall as a function of time and transferred the collected information to a personal computer to process into footfall patterns. The intra-rater reliability of this measure ranged from 0.77 to 0.99, while the inter-rater reliability ranged from 0.81 to 0.99.^[31]^ The GAITRite system recorded the gait velocity (cm/s), cadence (steps/min), step time of affected side and nonaffected side (s), and step length of affected side and nonaffected side (cm). For the measurements, participants were instructed to stand 3 m away from the electronic carpet and walk across it at a comfortable walking speed, stopping after walking 3 m past the electronic carpet. An examiner closely supervised each participant during the test to prevent falls. The measurements were repeated 3 times with a 1-minute break between each measurement to reduce the potential for bias due to muscle fatigue. The average of the 3 trials was calculated and recorded.^[29]^

#### 2.3.5. Fall efficacy

The Modified Falls Efficacy Scale was used for ****fall efficacy tests. Modified Falls Efficacy Scale consists of a total of 14 questions. Each item was scored on a 10-point visual analog scale, and the scores of all answered items were summed and adjusted to a range of 0 to 140 points. Higher scores indicate greater confidence and less fear of falling, while lower scores indicate less confidence and more fear of falling. The Modified Falls Efficacy Scale was chosen over other scales because its items include daily outdoor activities such as the use of public transport and road crossing. The Modified Falls Efficacy Scale demonstrated high internal consistency (Cronbach’s α = 0.95) and high test–retest reliability (intraclass correlation = 0.97).^[32]^

### 2.4. Statistical analysis

Data normality was assessed using the Kolmogorov–Smirnov test. Two-way ANOVA was used to compare the differences in balance, gait ability, and fall effects among the 3 groups at 3 different periods. One-way ANOVA was used for analysis of the data for comparison of differences within each group. If a significant interaction for a condition by side was found, post hoc analysis was performed to determine difference in pair-wise comparison. The statistical analysis was performed using SPSS 20.0 for Windows. The level of statistical significance was set at α ＝ 0.05.

## 3. Results

### 3.1. General characteristics of the subjects

Thirty patients with chronic stroke participated in this study. The patients were randomly divided into 3 groups (10 patients in each group). All patients in the 3 groups performed basic neurodevelopmental treatment. Group 1 performed balance exercises accompanied by gaze stability exercises. Group 2 performed gaze stability exercises and group 3 performed balance exercises. There were no significant differences in age, gender, weight, height, affected side, Korean-Mini-Mental State Examination, onset time, and stroke type among the 3 groups of subjects (*P* > .05). The general characteristics of the study subjects were as follows (Table 3).

**Table 3:** General characteristics of subjects

	Group 1 (n = 10)	Group 2 (n = 10)	Group 3 (n = 10)	*P*
	Mean ± SD	Mean ± SD	Mean ± SD	
Age (yr)	52.60 ± 17.83	51.60 ± 9.63	53.00 ± 12.77	.973
Gender (M/F)	7/3	7/3	6/4	.861
Height (cm)	170.90 ± 8.06	171.60 ± 8.77	168.40 ± 9.97	.707
Weight (kg)	67.60 ± 8.38	75.30 ± 10.94	67.80 ± 8.84	.135
K-MMSE (score)	26.88 ± 1.93	28.12 ± 1.76	27.13 ± 1.81	.873
Onset time (mo)	36.00 ± 14.58	36.20 ± 22.46	33.30 ± 11.92	.913
Stroke type (hemorrhage/infarction)	3/7	4/6	5/5	.659
Affected side (L/R)	4/6	4/6	5/5	.873

K-MMSE = Korean Version of Mini Status Examination, SD = standard deviation.

### 3.2. Changes in balance ability among the three groups

#### 3.2.1. Posture stability test with eyes open-overall stability index

##### 3.2.1.1. Changes in overall stability index with eyes open

When the eyes were opened, there were significant differences in the overall stability index according to the different time within the groups (*P* < .05). And there were significant differences among the 3 groups (*P* < .05). However, there was no significance of the interaction between group and time (*P* > .05).

##### 3.2.1.2. Comparison of overall stability index in each group and period of time with eyes open

There were significant differences in the overall stability index according to the different time within the group. In group 1, there was a significant difference between pre- and post-test, and between pre- and follow-up test. In group 2, there was a significant difference between pre- and post-test, and between pre- and follow-up test. In group 3, there was a significant difference between pre- and post-test, and between pre- and follow-up test (*P* < .05).

**Figure 2.**
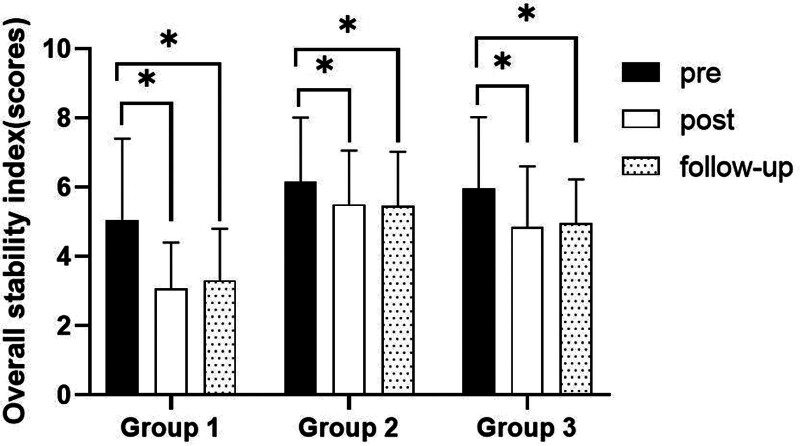
Comparison of overall stability index within each group. *Statistical significance within the group compared with the premeasurement (*P* < .05).

**Figure 3.**
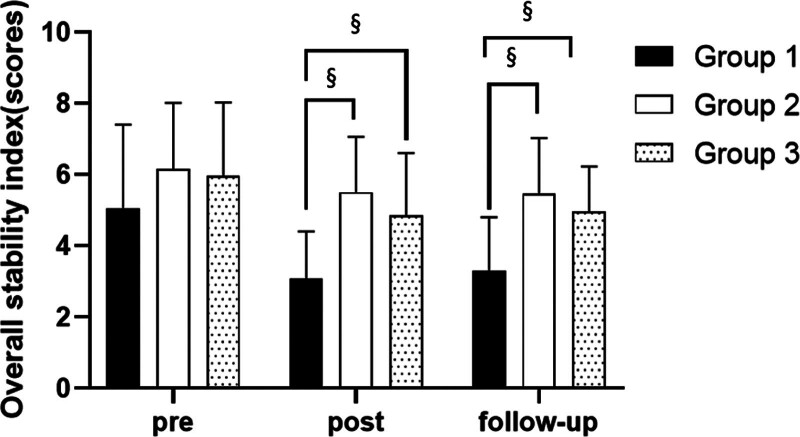
Comparison of overall stability index in each group. ^§^Statistical significance in the measurement outcomes between groups (*P* < .05).

At post-test period, there was a significant difference between group 1 and group 2 and between group 1 and group 3. At follow-up test period, there was a significant difference between group 1 and group 2 and between group 1 and group 3 (*P* < .05; Table 4; Figs. 2 and 3).

**Table 4:** Comparison of overall stability index in each group (eye open)

Group	Group 1 (n = 10)	Group 2 (n = 10)	Group 3 (n = 10)	*F*	*P*
	Mean ± SD	Mean ± SD	Mean ± SD		
Pre	5.06 ± 2.34	6.16 ± 1.85	5.95 ± 2.08	0.774	.471
Post	3.08 ± 1.32	5.50 ± 1.56	4.85 ± 1.75	6.480	.005*
Follow-up	3.31 ± 1.49	5.47 ± 1.56	4.96 ± 1.27	6.077	.007*
*F*	21.940	9.715	10.196		
*P*	.000*	.001*	.001*		

SD = standard deviation.

#### 3.2.2. Posture stability test with eyes close-overall stability index

##### 3.2.2.1. Changes in overall stability index with eyes close

When the eyes were closed, there were significant differences in the limit of stability test according to the different time within the groups. There were significant differences among the 3 groups. There was a significance of the interaction between group and time (*P* < .05).

##### 3.2.2.2. Comparison of overall stability index in each group and period of time with eyes close

There were significant differences in the overall stability index according to the different time within the groups. In group 1, there was a significant difference between pre- and post-test, and between pre- and follow-up test. In group 2, there was a significant difference between pre- and post-test, and between pre- and follow-up test. In group 3, there was a significant difference between pre- and post-test, and between pre- and follow-up test (*P* < .05).

**Figure 4.**
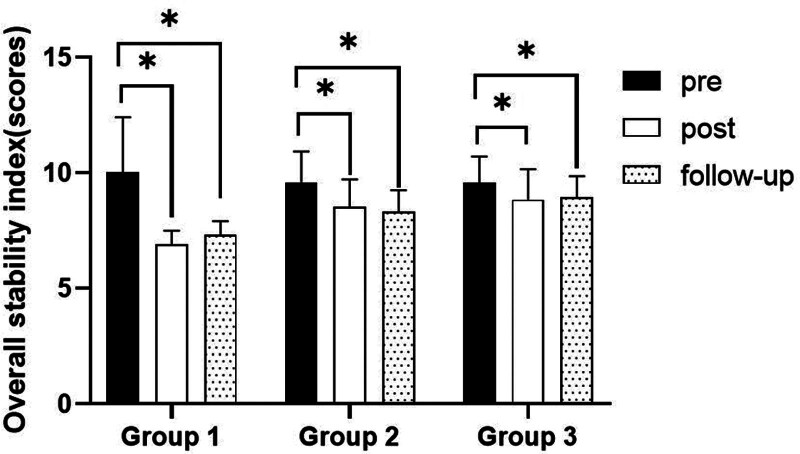
Comparison of overall stability index within each group. *Statistical significance within the group compared with the premeasurement (*P* < .05).

**Figure 5.**
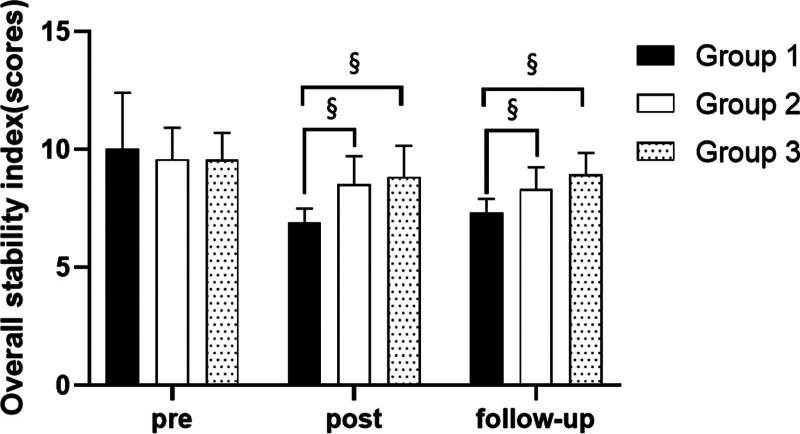
Comparison of overall stability index in each group. ^§^Statistical significance in the measurement outcomes between groups (*P* < .05).

At post-test period, there was a significant difference between group 1 and group 2 and between group 1 and group 3. At follow-up test period, there was a significant difference between group 1 and group 2 and between group 1 and group 3 (*P* < .05; Table 5; Figs. 4 and 5).

**Table 5:** Comparison of overall stability index in each group (eye close)

Group	Group 1 (n = 10)	Group 2 (n = 10)	Group 3 (n = 10)	*F*	*P*
	Mean ± SD	Mean ± SD	Mean ± SD		
Pre	10.03 ± 2.38	9.59 ± 1.34	9.56 ± 1.14	0.237	.790
Post	6.89 ± .62	8.52 ± 1.20	8.84 ± 1.31	9.242	.001*
Follow-up	7.32 ± .58	8.33 ± .92	8.96 ± .89	10.358	.000*
*F*	20.153	23.609	8.976		
*P*	.000*	.000*	.002*		

SD = standard deviation.

#### 3.2.3. Limit of stability test

##### 3.2.3.1. Changes in limit of stability test

There were significant differences in the limit of stability test according to the different time within the groups. There were significant differences among the 3 groups. There was a significance of the interaction between group and time (*P* < .05).

##### 3.2.3.2. Comparison of limit of stability test in each group and period of time

There were significant differences in the limit of stability test according to the different time within the groups. In group 1, there was a significant difference between pre- and post-test, and between pre- and follow-up test. In group 2, there was a significant difference between pre- and post-test, and between pre- and follow-up test. In group 3, there was a significant difference between pre- and post-test, and between pre- and follow-up test (*P* < .05).

**Figure 6.**
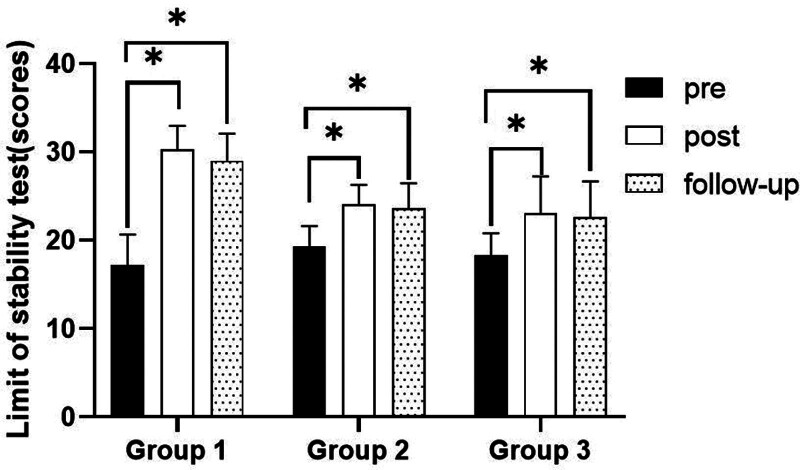
Comparison of limit of stability test within each group. *Statistical significance within the group compared with the premeasurement (*P* < .05).

**Figure 7.**
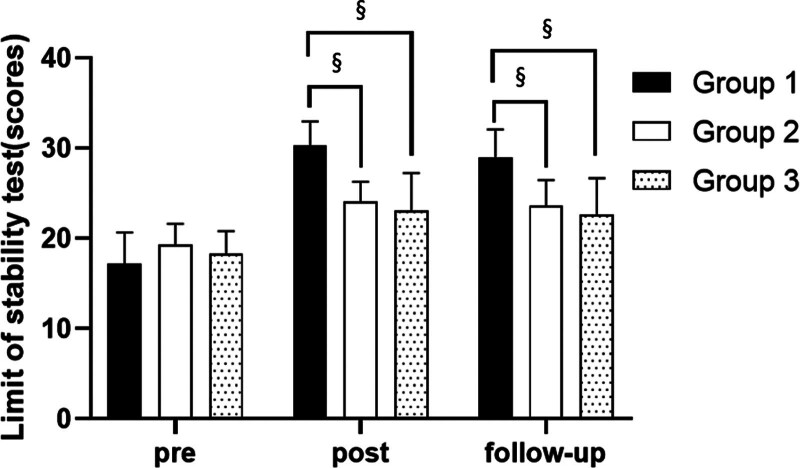
Comparison of limit of stability test in each group. ^§^Statistical significance in the measurement outcomes between groups (*P* < .05).

At post-test period, there was a significant difference between group 1 and group 2 and between group 1 and group 3. At follow-up test period, there was a significant difference between group 1 and group 2 and between group 1 and group 3 (*P* < .05; Table 6; Figs. 6 and 7).

**Table 6:** Comparison of limit of stability test in each group

Group	Group 1 (n = 10)	Group 2 (n = 10)	Group 3 (n = 10)	*F*	*P*
	Mean ± SD	Mean ± SD	Mean ± SD		
Pre	17.20 ± 3.49	19.30 ± 2.31	18.30 ± 2.50	1.393	.266
Post	30.30 ± 2.67	24.10 ± 2.18	23.10 ± 4.15	15.684	.000*
Follow-up	29.00 ± 3.09	23.60 ± 2.88	22.60 ± 4.09	11.996	.000*
*F*	297.342	35.513	44.658		
*P*	.000*	.000*	.000*		

SD = standard deviation.

### 3.3. Changes in gait ability among the three groups

#### 3.3.1. Gait velocity

##### 3.3.1.1. Changes in gait velocity

There were significant differences in the gait velocity according to the different time within the groups. And there were significant differences among the 3 groups (*P* < .05). However, there was no significance of the interaction between group and time (*P* > .05).

##### 3.3.1.2. Comparison of gait velocity in each group and period of time

There were significant differences in the gait velocity according to the different time within the groups. In the group 1, there was a significant difference between pre- and post-test, and between pre- and follow-up test. In group 2, there was a significant difference between pre- and post-test, and between pre- and follow-up test. In group 3, there was a significant difference between pre- and post-test, and between pre- and follow-up test (*P* < .05).

**Figure 8.**
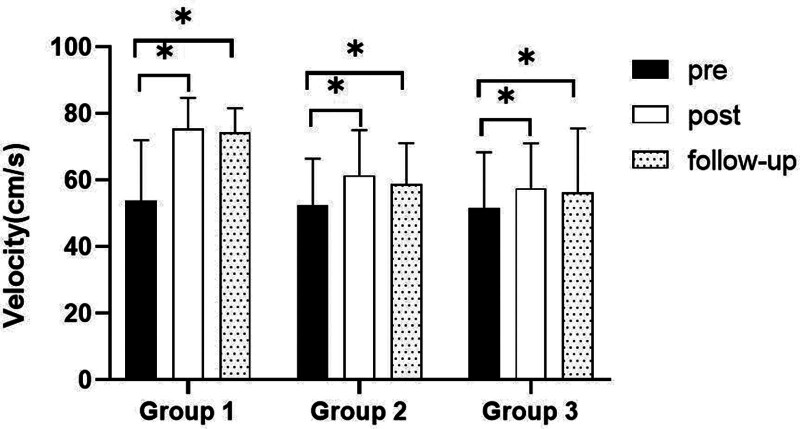
Comparison of gait velocity within each group. *Statistical significance within the group compared with the premeasurement (*P* < .05).

**Figure 9.**
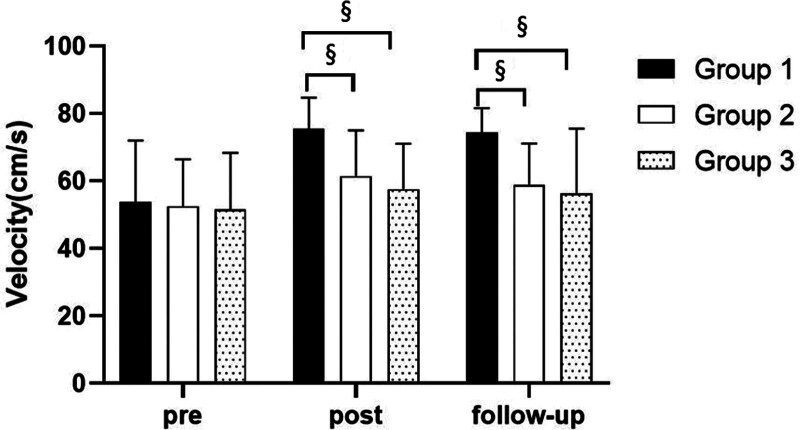
Comparison of gait velocity in each group. ^§^Statistical significance in the measurement outcomes between groups (*P* < .05).

At post-test period, there was a significant difference between group 1 and group 2 and between group 1 and group 3. At follow-up test period, there was a significant difference between group 1 and group 2 and between group 1 and group 3 (*P* < .05; Table 7; Figs. 8 and 9).

**Table 7:** Comparison of gait velocity in each group

Group	Group 1 (n = 10)	Group 2 (n = 10)	Group 3 (n = 10)	*F*	*P*
	Mean ± SD	Mean ± SD	Mean ± SD		
Pre	53.78 ± 18.23	52.52 ± 13.91	51.57 ± 16.82	0.035	.966
Post	75.37 ± 9.34	61.45 ± 13.61	57.55 ± 13.47	5.798	.008*
Follow-up	74.37 ± 7.27	58.88 ± 12.24	56.19 ± 19.35	5.004	.014*
*F*	23.117	19.417	5.341		
*P*	.000*	.000*	.015*		

SD = standard deviation.

#### 3.3.2. Cadence

##### 3.3.2.1. Changes in cadence

There were significant differences in the cadence according to the different time within the groups. There were significant differences among the 3 groups (*P* < .05). However, there was no significance of the interaction between group and time (*P* > .05).

##### 3.3.2.2. Comparison of cadence in each group and period of time

There were significant differences in the cadence according to the different time within the groups. In group 1, there was a significant difference between pre- and post-test, and between pre- and follow-up test. In group 2, there was a significant difference between pre- and post-test, and between pre- and follow-up test. In the group 3, there was a significant difference between pre- and post-test, and between pre- and follow-up test (*P* < .05).

**Figure 10.**
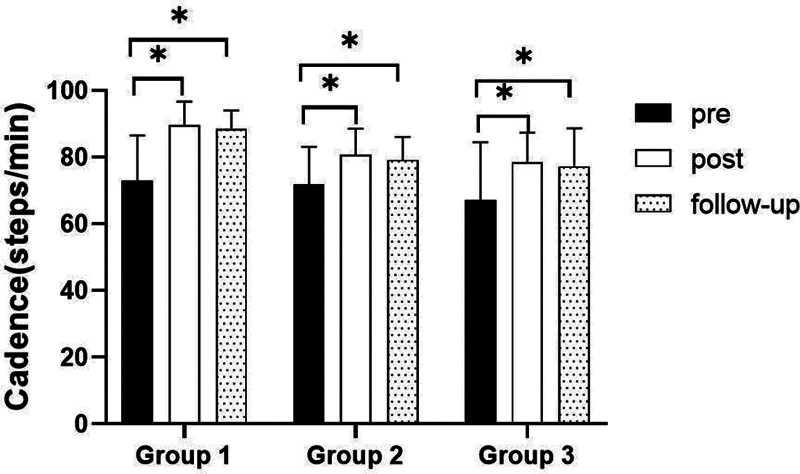
Comparison of cadence within each group. *Statistical significance within the group compared with the premeasurement (*P* < .05).

**Figure 11.**
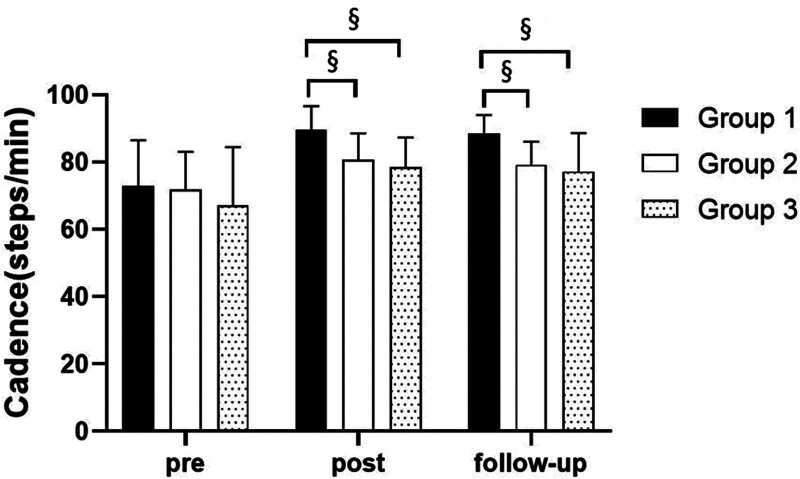
Comparison of cadence in each group. ^§^Statistical significance in the measurement outcomes between groups (*P* < .05).

At post-test period, there was a significant difference between group 1 and group 2 and between group 1 and group 3. At follow-up test period, there was a significant difference between group 1 and group 2 and between group 1 and group 3 (*P* < .05; Table 8; Figs. 10 and 11).

**Table 8:** Comparison of cadence in each group

Group	Group 1 (n = 10)	Group 2 (n = 10)	Group 3 (n = 10)	*F*	*P*
	Mean ± SD	Mean ± SD	Mean ± SD		
Pre	72.94 ± 13.64	71.88 ± 11.23	67.15 ± 17.39	0.413	.665
Post	89.71 ± 6.97	80.77 ± 7.85	78.49 ± 8.90	5.568	.009*
Follow-up	88.59 ± 5.43	79.24 ± 6.87	77.27 ± 11.40	5.311	.011*
*F*	20.676	13.355	8.532		
*P*	.000*	.000*	.002*		

SD = standard deviation.

#### 3.3.3. Step length on affected side

##### 3.3.3.1. Changes in step length on affected side

There were significant differences in the step length on affected side according to the different time within the groups. There were significant differences among the 3 groups (*P* < .05). However, there was no significance of the interaction between group and time (*P* > .05).

##### 3.3.3.2. Comparison of step length on affected side in each group and period of time

There were significant differences in the step length on affected side according to the different time within the groups. In group 1, there was a significant difference between pre- and post-test, and between pre- and follow-up test. In group 2, there was a significant difference between pre- and post-test, and between pre- and follow-up test. In group 3, there was a significant difference between pre- and post-test, and between pre- and follow-up test (*P* < .05).

**Figure 12.**
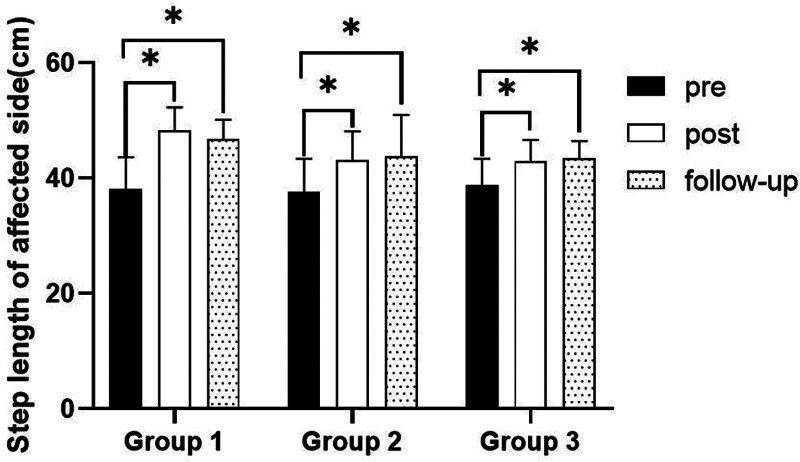
Comparison of step length on affected side within each group. *Statistical significance within the group compared with the premeasurement (*P* < .05).

**Figure 13.**
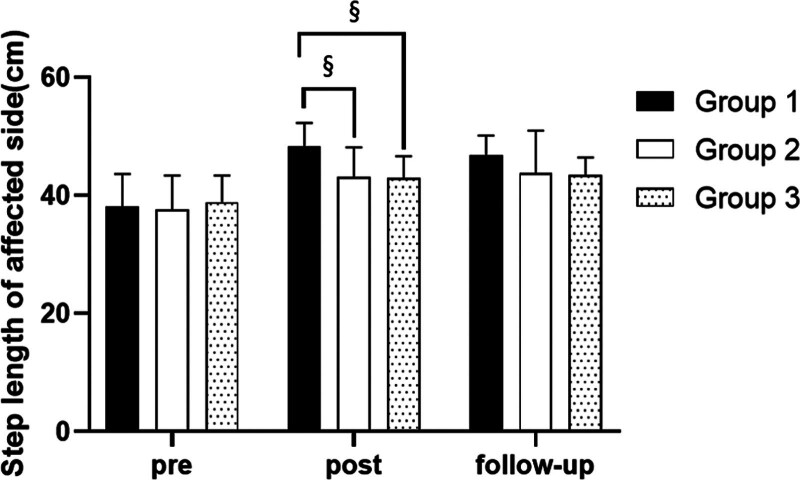
Comparison of step length on affected side in each group. ^§^Statistical significance in the measurement outcomes between groups (*P* < .05).

At post-test period, there was a significant difference between group 1 and group 2 and between group 1 and group 3 (*P* < .05; Table 9; Figs. 12 and 13).

**Table 9:** Comparison of step length on affected side in each group

Group	Group 1 (n = 10)	Group 2 (n = 10)	Group 3 (n = 10)	*F*	*P*
	Mean ± SD	Mean ± SD	Mean±SD		
Pre	38.08 ± 5.56	37.58 ± 5.79	38.90 ± 4.46	0.158	.855
Post	48.21 ± 4.07	43.11 ± 5.04	42.91 ± 3.70	4.861	.016*
Follow-up	46.73 ± 3.38	43.73 ± 7.23	43.46 ± 2.95	1.369	.271
*F*	38.612	11.307	9.146		
*P*	.000*	.001*	.002*		

SD = standard deviation.

#### 3.3.4. Step length on nonaffected side

##### 3.3.4.1. Changes in step length on nonaffected side

There were significant differences in the step length on nonaffected side according to the different time within the groups. There were significant differences among the 3 groups (*P* < .05). However, there was no significance of the interaction between group and time (*P* > .05).

##### 3.3.4.2. Comparison of step length on nonaffected side in each group and period of time

There were significant differences in the cadence according to the different time within the groups. In group 1, there was a significant difference between pre- and post-test, and between pre- and follow-up test. In group 2, there was a significant difference between pre- and post-test. In group 3, there was a significant difference between pre- and post-test, and between pre- and follow-up test (*P* < .05).

**Figure 14.**
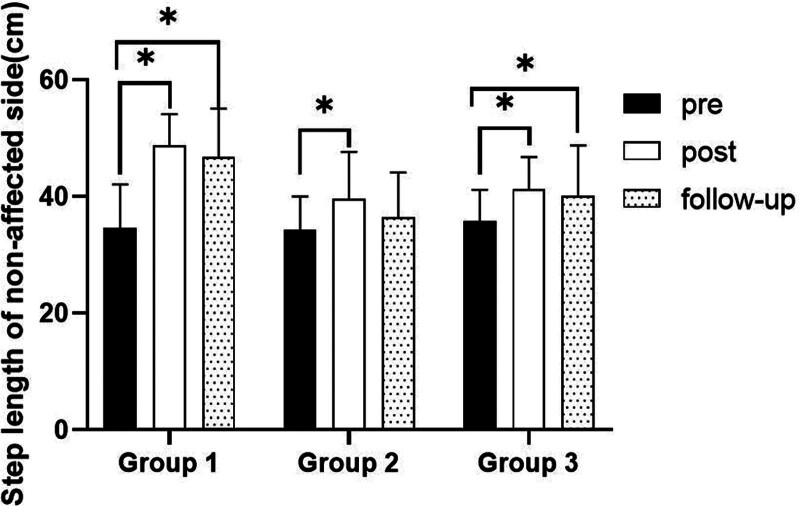
Comparison of step length on nonaffected side within each group. *Statistical significance within the group compared with the premeasurement (*P* < .05).

**Figure 15.**
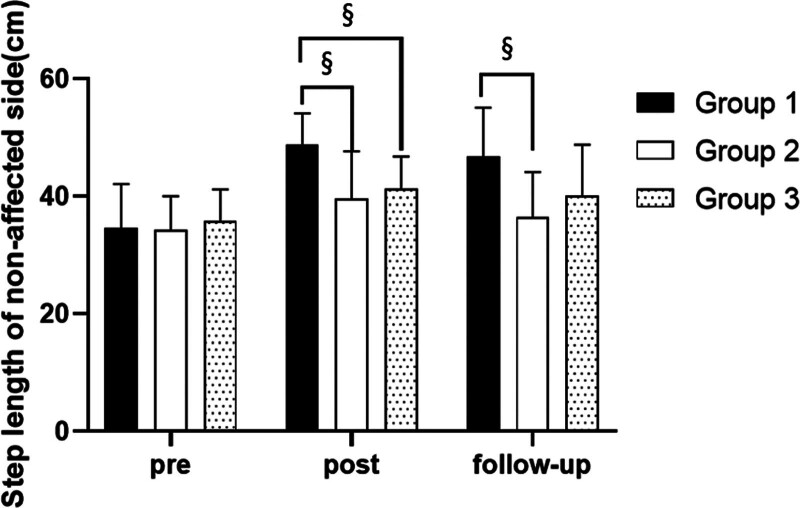
Comparison of step length on nonaffected side in each group. ^§^Statistical significance in the measurement outcomes between groups (*P* < .05).

At post-test period, there was a significant difference between group 1 and group 2 and between group 1 and group 3. At follow-up test period, there was a significant difference between group 1 and group 2 (*P* < .05; Table 10; Figs. 14 and 15).

**Table 10:** Comparison of step length on nonaffected side in each group

Group	Group 1 (n = 10)	Group 2 (n = 10)	Group 3 (n = 10)	*F*	*P*
	Mean ± SD	Mean ± SD	Mean ± SD		
Pre	34.68 ± 7.38	34.25 ± 5.79	35.77 ± 5.40	0.158	.855
Post	48.70 ± 5.40	39.63 ± 8.04	41.30 ± 5.47	5.654	.009*
Follow-up	46.78 ± 8.30	36.43 ± 7.71	40.15 ± 8.60	4.073	.028*
*F*	29.379	4.713	13.968		
*P*	.000*	.023*	.000*		

SD = standard deviation.

#### 3.3.5. Step time on affected side

##### 3.3.5.1. Changes in step time on affected side

There were significant differences in the step time on affected side according to the different time within the groups. There were significant differences among the 3 groups. There was a significance of the interaction between group and time (*P* < .05).

##### 3.3.5.2. Comparison of step time on affected side in each group and period of time

There were significant differences in the step time on affected side according to the different time within the groups. In group 1, there was a significant difference between pre- and post-test, and between pre- and follow-up test. In group 2, there was a significant difference between pre- and post-test, and between pre- and follow-up test. In group 3, there was a significant difference between pre- and post-test, and between pre- and follow-up test (*P* < .05).

**Figure 16.**
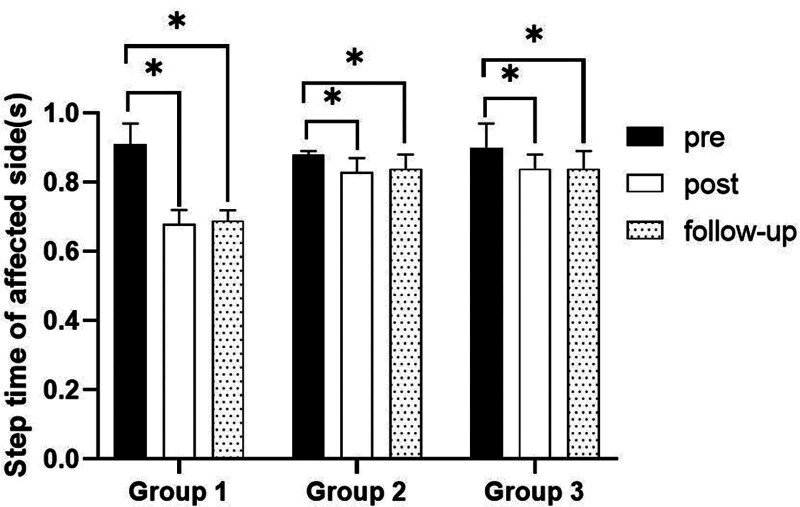
Comparison of step time on affected side within each group. *Statistical significance within the group compared with the premeasurement (*P* < .05).

**Figure 17.**
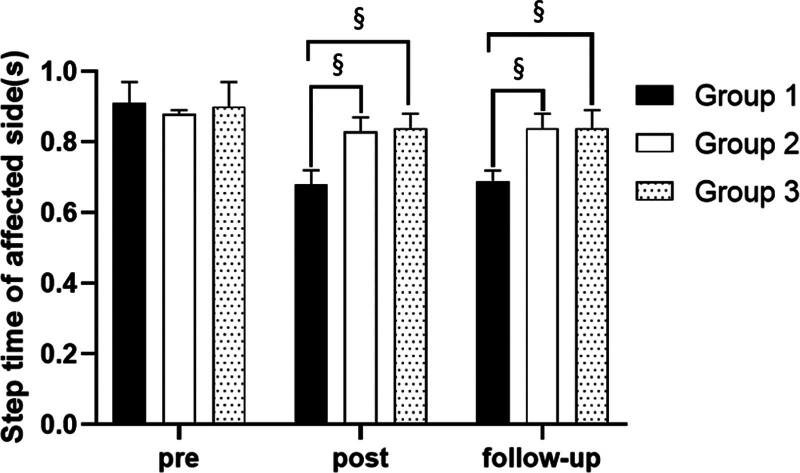
Comparison of step time on affected side in each group. ^§^Statistical significance in the measurement outcomes between groups (*P* < .05).

At post-test period, there was a significant difference between group 1 and group 2 and between group 1 and group 3. At follow-up test period, there was a significant difference between group 1 and group 2 and between group 1 and group 3 (*P* < .05; Table 11; Figs. 16 and 17).

**Table 11:** Comparison of step time on affected side in each group

Group	Group 1 (n = 10)	Group 2 (n = 10)	Group 3 (n = 10)	*F*	*P*
	Mean ± SD	Mean ± SD	Mean ± SD		
Pre	0.91 ± 0.06	0.88 ± 0.01	0.90 ± 0.07	1.010	.378
Post	0.68 ± 0.04	0.83 ± 0.04	0.84 ± 0.04	48.489	.000*
Follow-up	0.69 ± 0.03	0.84 ± 0.04	0.84 ± 0.05	48.250	.000*
*F*	173.466	14.799	10.601		
*P*	.000*	.000*	.001*		

SD = standard deviation.

#### 3.3.6. Step time on nonaffected side

##### 3.3.6.1. Changes in step time on nonaffected side

There were significant differences in the step time on nonaffected side according to the different time within the groups. There were significant differences among the 3 groups (*P* < .05). However, there was no significance of the interaction between group and time (*P* > .05).

##### 3.3.6.2. Comparison of step time on nonaffected side in each group and period of time

There were significant differences in the step time on nonaffected side according to the different time within the groups. In group 1, there was a significant difference between pre- and post-test, and between pre- and follow-up test. In group 2, there was a significant difference between pre- and post-test, and between pre- and follow-up test. In group 3, there was a significant difference between pre- and post-test, and between pre- and follow-up test (*P* < .05).

**Figure 18.**
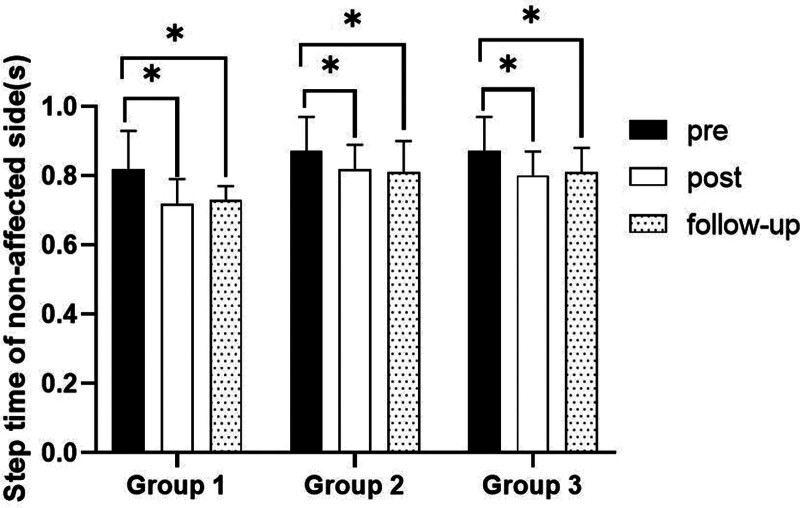
Comparison of step time on nonaffected side within each group. *Statistical significance within the group compared with the premeasurement (*P* < .05).

**Figure 19.**
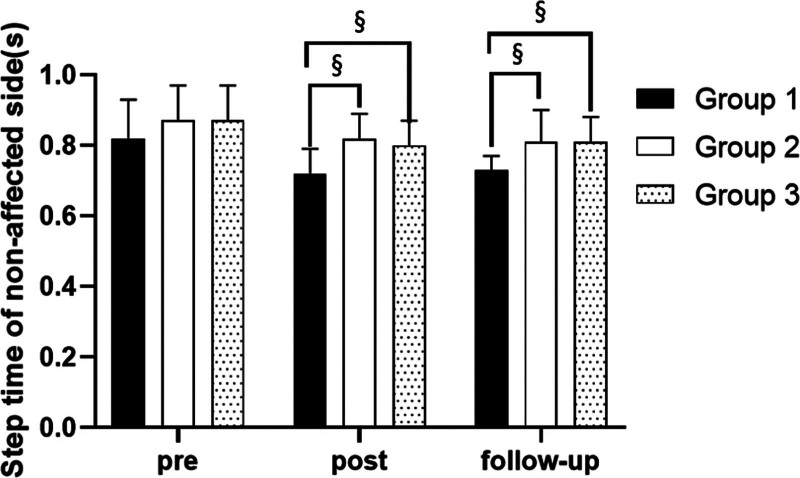
Comparison of step time on nonaffected side in each group. ^§^Statistical significance in the measurement outcomes between groups (*P* < .05).

At post-test period, there was a significant difference between group 1 and group 2 and between group 1 and group 3. At follow-up test period, there was a significant difference between group 1 and group 2 and between group 1 and group 3 (*P* < .05; Table 12; Figs. 18 and 19).

**Table 12:** Comparison of step time on nonaffected side in each group

Group	Group 1 (n = 10)	Group 2 (n = 10)	Group 3 (n = 10)	*F*	*P*
	Mean ± SD	Mean ± SD	Mean ± SD		
Pre	0.82 ± 0.11	0.87 ± 0.10	0.87 ± 0.10	0.871	.430
Post	0.72 ± 0.07	0.82 ± 0.07	0.80 ± 0.07	5.450	.010*
Follow-up	0.73 ± 0.04	0.81 ± 0.09	0.81 ± 0.07	4.951	.015*
*F*	12.412	11.599	14.869		
*P*	.000*	.001*	.000*		

SD = standard deviation.

### 3.4. TUG test

#### 3.4.1. Changes in TUG test

There were significant differences in the time up and go test according to the different time within the groups. There were significant differences among the 3 groups (*P* < .05). However, there was no significance of the interaction between group and time (*P* > .05).

#### 3.4.2. Comparison of TUG test in each group and period of time

There were significant differences in the time up and go test according to the different time within the groups. In group 1, there was a significant difference between pre- and post-test, and between pre- and follow-up test. In group 2, there was a significant difference between pre- and post-test, and between pre- and follow-up test. In group 3, there was a significant difference between pre- and post-test, and between pre- and follow-up test (*P* < .05).

**Figure 20.**
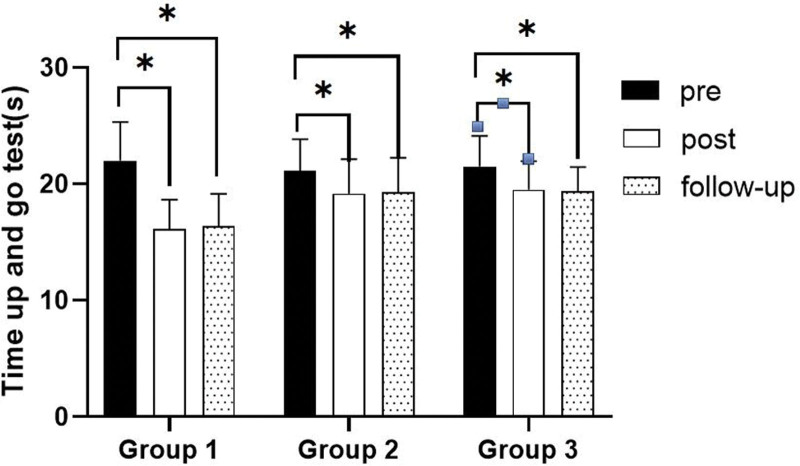
Comparison of time up and go test within each group. *Statistical significance within the group compared with the premeasurement (*P* < .05).

**Figure 21.**
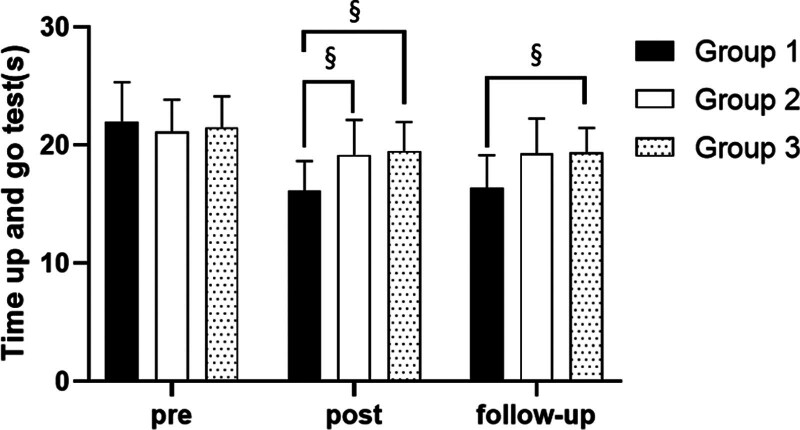
Comparison of time up and go test in each group. ^§^Statistical significance in the measurement outcomes between groups (*P* < .05).

At post-test period, there was a significant difference between group 1 and group 2 and between group 1 and group 3. At follow-up test period, there was a significant difference between group 1 and group 3 (*P* < .05; Table 13; Figs. 20 and 21).

**Table 13:** Comparison of time up and go test in each group

Group	Group 1 (n = 10)	Group 2 (n = 10)	Group 3 (n = 10)	*F*	*P*
	Mean ± SD	Mean ± SD	Mean ± SD		
Pre	22.00 ± 3.33	21.10 ± 2.77	21.50 ± 2.64	0.237	.790
Post	16.10 ± 2.56	19.20 ± 2.94	19.50 ± 2.46	5.009	.014*
Follow-up	16.40 ± 2.76	19.30 ± 2.95	19.40 ± 2.07	4.240	.025*
*F*	83.521	21.000	16.546		
*P*	.000*	.000*	.000*		

SD = standard deviation.

### 3.5. Berg Balance Scale

#### 3.5.1. Changes in BBS

There were significant differences in the BBS according to the different time within the groups (*P* < .05). However, there were no significant differences among the 3 groups and there was no significance of the interaction between group and time (*P* > .05).

#### 3.5.2. Comparison of BBS in each group and period of time

There were significant differences in the BBS according to the different time within the groups. In group 1, there was a significant difference between pre- and post-test, and between pre- and follow-up test. In group 2, there was a significant difference between pre- and post-test, and between pre- and follow-up test. In group 3, there was a significant difference between pre- and post-test, and between pre- and follow-up test (*P* < .05).

**Figure 22.**
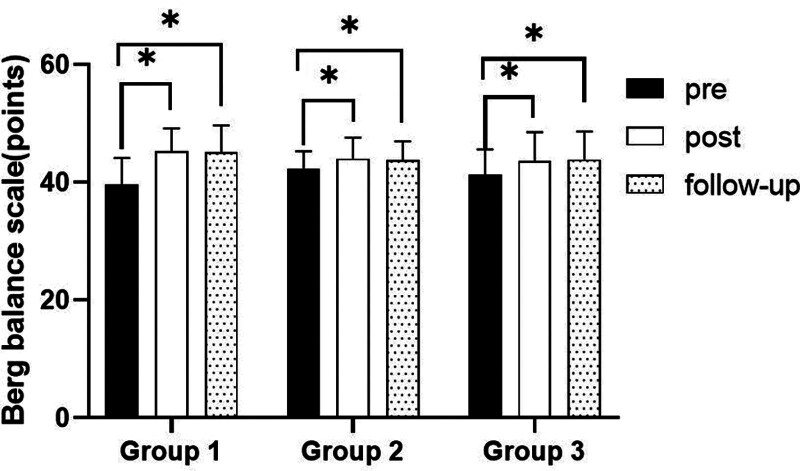
Comparison of berg balance scale within each group. *Statistical significance within the group compared with the premeasurement (*P* < .05).

**Figure 23.**
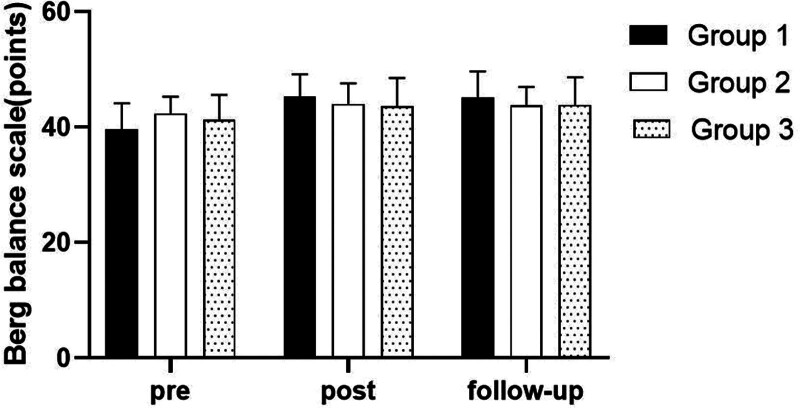
Comparison of berg balance scale in each group.

However, there were no significant differences among the 3 groups at any time (*P* > .05; Table 14; Figs. 22 and 23).

**Table 14:** Comparison of berg balance scale in each group

Group	Group 1 (n = 10)	Group 2 (n = 10)	Group 3 (n = 10)	*F*	*P*
	Mean ± SD	Mean ± SD	Mean ± SD		
Pre	39.60 ± 4.50	42.40 ± 2.88	41.20 ± 4.37	1.244	.304
Post	45.30 ± 3.86	44.00 ± 3.59	43.60 ± 4.90	0.457	.638
Follow-up	45.10 ± 4.56	43.80 ± 3.16	43.90 ± 4.75	0.295	.747
*F*	44.772	8.143	9.491		
*P*	.000*	.003*	.002*		

SD = standard deviation.

### 3.6. Modified falls efficacy scale

#### 3.6.1. Changes in Modified Falls Efficacy Scale

There were significant differences in the Modified Falls Efficacy Scale according to the different time within the groups (*P* < .05). However, there were no significant differences among the 3 groups and there was no significance of the interaction between group and time (*P* > .05).

#### 3.6.2. Comparison of Modified Falls Efficacy Scale in each group and period of time

There were significant differences in the Modified Falls Efficacy Scale according to the different time within the groups. In group 1, there was a significant difference between pre- and post-test, and between pre- and follow-up test. In group 2, there was a significant difference between pre- and post-test, and between pre- and follow-up test. In group 3, there was a significant difference between pre- and post-test, and between pre- and follow-up test (*P* < .05).

**Figure 24.**
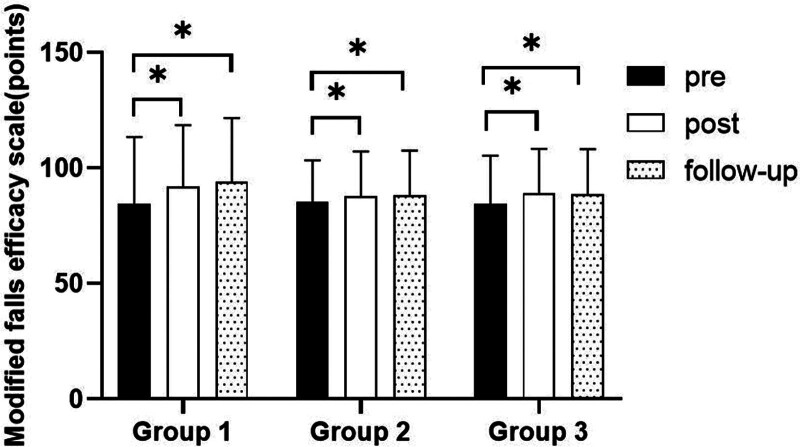
Comparison of modified falls efficacy scale within each group. *Statistical significance within the group compared with the premeasurement (*P* < .05).

**Figure 25.**
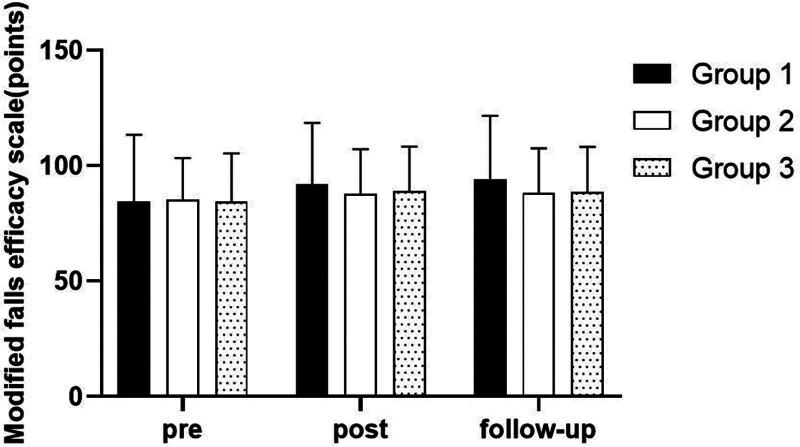
Comparison of modified falls efficacy scale in each group.

However, there were no significant differences among the 3 groups at any time (*P* > .05; Table 15; Figs. 24 and 25).

**Table 15:** Comparison of modified falls efficacy scale in each group

Group	Group 1 (n = 10)	Group 2 (n = 10)	Group 3 (n = 10)	*F*	*P*
	Mean ± SD	Mean ± SD	Mean ± SD		
Pre	84.50 ± 28.98	85.30 ± 18.06	84.60 ± 20.77	0.004	.996
Post	92.10 ± 26.47	87.80 ± 19.39	89.00 ± 19.35	0.102	.904
Follow-up	94.10 ± 27.58	88.10 ± 19.43	88.60 ± 19.56	0.219	.805
*F*	27.551	11.374	23.715		
*P*	.000*	.001*	.000*		

SD = standard deviation.

## 4. Discussion

This study aimed to examine the effect of gaze stability exercises on balance, gait ability, and fall efficacy in patients with chronic stroke, as well as to investigate whether any observed effects were maintained 2 weeks later.

This study used Biodex Balance System to test the overall stability index with their eyes open or closed and limit of stability of patients standing in an upright position on a circular platform. These variables can intuitively detect the changes in balance ability. The study found significant differences in balance ability variables across different time points within the 3 groups. There were significant improvements in balance ability within each group from pre to post-test and from pretest to follow-up. Both the overall stability index and limit of stability showed significant changes, with a decrease in the former and an increase in the latter during post-test and follow-up compared with pretest. These changes indicate improved balance ability. Although no significant difference was observed between post-test and follow-up, suggesting maintenance of balance ability post-experiment. Group 1 showed significantly greater improvement in balance ability compared to groups 2 and 3 during both post-test and follow-up periods, indicating its superiority and effectiveness in enhancing balance ability.

The BBS was used to evaluate balance-related disorders in this study. The results of this study showed that there were significant differences over the different time within the 3 groups in BBS. In all groups, there was a significant difference between pre- and post-test, and between pre- and follow-up test. However, there was no significant difference between post and follow-up test, indicating that the patient’s balance ability could be maintained after the experiment. In the comparison of the BBS among 3 groups, it was found that there were no significant differences among 3 group. The reason for this may be that the evaluation of BBS is more subjective, or the experiment time is too short.

Balance is highly complex, involving the stimulation of the central nervous system through visual, auditory, vestibular, and proprioceptive sense inputs to respond quickly and accurately to environmental changes.^[33]^ Stroke patients often experience balance disorders and may have difficulty properly utilizing vestibular information. Their balance and gait functions primarily based on visual input.^[34]^ Balance training is considered to be an important component of fall prevention programs and has been shown to enhance various abilities necessary for postural control.^[35]^ Previous reviews reported that balance training facilitate a strong beneficial effect on balance, walking ability, personal activities of daily living, and balance skills in patients with stroke.^[14]^ In this study, group 3 underwent a 4-week balance training. Stroke patients showed a significant increase in their balance ability in the post-test period and in follow-up tests after 2 weeks compared with pretest period. Even in follow-up tests after 2 weeks, the patient’s balance ability was well maintained. The results of this study are consistent with the above positive effects of balance training on stroke patients.

Vision is an important sensory signal for adapting to the external environment, processing information necessary for perception, localization, and proprioception through voluntary or involuntary eye movements.^[36]^ Gaze stability is necessary to coordinate the movements of the head, trunk, and pelvis during gait.^[37]^ After a stroke, individuals often exhibit abnormal coordination between axial segments and pelvic rotations when turning their heads, which affects their balance during gait. Furthermore, the decreased stability of the trunk and head after a stroke can result in lower-quality visual information, further impacting balance negatively.^[2,38]^ Gaze stability exercises involve actively moving the head while focusing on a target for a specified duration, which is a method of vestibular rehabilitation. This stimulates nerves in the brainstem and cerebellum through repetitive activation of the vestibulo-ocular and VSRs, promoting the recovery of vestibular function.^[39]^ The VOR is the first mechanism of gaze stability. During head movement, the VOR stabilizes gaze (the position of the eyes in space) by generating eye movements at the same speed and opposite direction to head movements, thereby allowing for clear vision. In contrast, the VSR maintains postural stability by activating contractions of anti-gravity muscles to counteract gravity.^[2,40,41]^ The VOR, a reflex induced by the vestibular system, is involved in maintaining balance and equilibrium by playing an important role in recognizing body position in space.^[42,43]^ When tilting or rotating the head in an upright position, the semicircular canals and otoliths are stimulated, leading to activation of the vestibular nerve and nuclei. Subsequently, some of these stimuli are transmitted to the spinal cord via the vestibule-spinal tracts, inducing muscle contractions to maintain the upright position. This collective response is known as the VSR, which is important for maintaining an upright posture.^[22,44]^ Pimenta et al^[2]^ reported that during eye movement, vestibular receptor information is transmitted through the vestibular nucleus to the oculomotor nucleus due to VOR, which stabilizes vision through contraction of the extraocular eye muscles. The information from the vestibular receptor through the vestibular nerve nucleus causes tension contraction of the antigravitational muscle through the vestibular spinal nerve tract to maintain stability between the bodies. In the research of Bae^[45]^, the area, distance, and speed of center of pressure in the static balance evaluation were significantly reduced, and these results demonstrated that eye movement stimulated the muscles of the eyeball, thus having a positive effect on the static balance. The research results of this experiment are consistent with the content mentioned above, proving that eye movement improves the balance ability of stroke patients. In this study, both group 1 and group 2 underwent gaze stability exercise, and balance ability in both groups increased significantly before and after the experiment. Group 3 also experienced a significant increase in balance ability through balance exercise. But compared to group 2 and group 3, group 1 was more effective at improving balance ability. The reason for this may be that group 1 performed eye stability exercises while doing balance training. When balance exercise and gaze stability exercise are performed at the same time, the body shake and instability are greater than the balance exercise or gaze stability exercise alone, and the body is more difficult to achieve stability, and the balance requirements are higher.

The GAITRite system was used to record the gait velocity, cadence, step time of affected side and nonaffected side, and step length of affected side and nonaffected side. For step length of affected side, there were significant increased between pre- and post-test, and between pre- and follow-up test in all groups. In the post-test period, group 1 increased more step length of affected side than group 2 and group 3. For step length of nonaffected side, there were significant increased between pre- and post-test and between pre- and follow-up test in group 1 and group 3, and there were significant increased between pre- and post-test in group 2. In the post-test period, group 1 increased more step length of nonaffected side than group 2 and group 3. In follow-up test period, group 1 increased more step length of nonaffected side than group 2. For gait velocity, cadence, step time of affected side and nonaffected side, there were significant differences between pre- and post-test, and between pre- and follow-up test. In the comparison of the gait ability among 3 groups, it was found that group 1 was improved more than that of group 2 and group 3 during the post-test period and follow-up test period. When all variables were compared among the 3 groups, it can be found that group 1 is better and more effective in improving gait ability than group 2 and group 3.

After a stroke, many patients experience asymmetrical posture and abnormal balance, leading to difficulties in standing and gait.^[43]^ Additionally, post-stroke, reduced control over body and gaze movements can impair performance during gait and turning.^[46,47]^ Gaze stability is necessary to coordinate the movements of the head, trunk, and pelvis during gait.^[37]^ Vision is one of the important factors in the rehabilitation of walking abilities.^[48]^ The lower the accuracy of vision, the more incorrect visual information can lead to a decrease in walking abilities, leading to problems with independent activities in daily life and ultimately affecting society.^[19,49]^ Previous studies on weight distribution and gait in stroke patients have reported the effects of visual training. Similarly, research that included behavioral observation training using visual information for stroke patients reported improvements in weight bearing, stability, and gait function.^[43,50]^ Tramontano et al^[34]^ reported that 4 weeks of eye movement exercise improved walking ability, such as walking speed, in patients with acute stroke. Kang and Yu^[43]^ reported significant differences in stride speed, stride length, and steps per minute after using cards and tools for eye movement project arbitration in stroke patients. A study aimed at identifying factors affecting gait in community-based stroke patients investigated balance, reduced cardiopulmonary function, and gait speed. The study reported that a decline in balance leads to a deterioration in gait function.^[51]^ Zampieri and Di Fabio^[52]^ explained that direct stimulation of visual and vestibular organ improved balance ability and brought positive changes to walking ability. And reports that an eye movement program can improve stance phase time, step time, and more.^[52]^ Additionally, exercise programs utilizing visual feedback in stroke patients have been reported to improve gait speed, muscle strength, and balance ability, supporting the importance of visual training.^[53,54]^ In stroke patients with difficulties in gait and balance, the application of eye movement training may directly stimulate the visual and vestibular systems, thereby enhancing balance ability and potentially leading to positive changes in gait function.^[55]^ In this study, each group significantly increased their gait ability under the effects of gaze stability exercise and balance exercise. But compared to group 2 and group 3, group 1 was more effective at improving gait ability. The reason for this may be that group 1 improves balance better than group 2 and group 3, resulting in group 1 having a greater improvement in gait ability than the other 2 groups. These findings suggest that gaze stability exercises can significantly improve the gait ability of stroke patients.

In this study, the TUG test was used to assess gait ability. There were significant decreased in walking time between pre- and post-test, and between pre- and follow-up test in all group. However, there was no significant difference between post and follow-up test, indicating that the patient’s gait ability could be maintained after the experiment. In the post-test period and follow-up test period, the walking time of group 1 more decrease than group 2 and group 3. Therefore, it can be found that group 1 is better and more effective in improving gait ability than group 2 and group 3.

Pimenta et al^[2]^ reported that compared with the conventional physical therapy group, there was a significant improvement in TUG test in the study of gaze stability exercise in stroke patients, which is consistent with the research results reported in this experimental report. In addition, significant changes in standing up and walking examinations are positively correlated with changes in walking ability, so I believe walking ability has improved. This is thought to be the result of visual stimuli being transmitted through the central nervous system and integrated with other sensory stimuli, leading the muscles to make the correct anticipatory adjustment.^[56,57]^ In this study, each group significantly decreased their walking time under the effects of gaze stability exercise and balance exercise. But compared to group 2 and group 3, group 1 was more effective at improving gait ability. The reason for this may be that group 1 improves balance better than group 2 and group 3, resulting in group 1 having a greater improvement in gait ability than the other 2 groups.

The Modified Falls Efficacy Scale was used to evaluate fear of falling in this study. The results of this study showed that there were significant differences according to the different time within the 3 groups in BBS. In all group, there was a significant difference between pre- and post-test, and between pre- and follow-up test. However, there was no significant difference between post and follow-up test, indicating that the patient’s balance ability could be maintained after the experiment. In the comparison of the Modified Falls Efficacy Scale among 3 groups, it was found that there were no significant differences among 3 groups. The reason for this may be that the evaluation of Modified Falls Efficacy Scale is more subjective, or the experiment time is too short.

According to research on neurological disorders, stroke patients have an increased risk of falling due to various impairments in motor, sensory, and higher brain functions.^[58]^ So they tend to avoid moving quickly to prevent falls. In stroke, physical limitations and psychological fear can secondarily reduce vestibular function.^[39]^ The experience of a fall is associated with increased fear of falling, loss of confidence, restriction of activity, becoming dependent on performing daily activities, and decreased quality of life.^[59]^ In Zahedian-Nasab’s study^[60]^, the impact of applying virtual reality sports for an average age of 81 years on the sense of fall injury efficacy was studied. The experimental results showed that virtual reality sports brought about an improvement in balance ability. The improvement of balance ability reduces the fear of falling injuries and enhances the sense of effectiveness of falling injuries. This is consistent with the improvement of balance ability caused by eye movement planning in this study, which increases the sense of fall efficacy.^[60]^ Due to the significant improvement in balance ability in all 3 groups, there was also a significant increase in Modified Falls Efficacy Scale among the 3 groups. As for the lack of significant differences between the 3 groups, it may be due to the evaluation of Modified Falls Efficacy Scale is more subjective, or the experiment time is too short.

Our study has some important limitations. First, at the beginning of the study, there were many potential participants, but the number decreased due to strict inclusion/exclusion criteria. Although the sample size was defined based on power analysis results, it turned out to be relatively small. It can be speculated that increasing the sample size would likely result in a greater number of significant differences. Second, considering that these research findings apply specifically to individuals in the chronic phase after stroke, it is challenging to generalize the results to all stroke patients. Therefore, future researchers should evaluate the effects of gaze stability exercises on patients with moderate to severe physical impairments in the acute and subacute phases of stroke. Third, due to safety considerations, in order to avoid falling accidents during the experiment, there are certain limitations in the evaluation, which cannot be evaluated in a completely independent state. Such as swaying during balance assessments, or partial intervention to prevent falls during TUG test. Therefore, we should take this into account in future experiments to avoid interference from external factors.

## 5. Conclusion

This study investigated the impact of gaze stability exercises on balance, gait, and fall efficacy in chronic stroke patients, along with the maintenance of effects over 2 weeks. Results showed significant improvements in balance, gait ability, and fall efficacy across the groups, with sustained effects observed in follow-up tests. Group 1, which combined balance exercises and gaze stability exercises, showed greater improvement than the other 2 groups in all outcomes except for the BBS, Modified Falls Efficacy Scale, and TUG test. Thus, integrating gaze stability exercises with neurodevelopmental treatment proves effective in enhancing balance and gait among stroke patients, suggesting its recommendation as a comprehensive intervention strategy.

## Author contributions

**Conceptualization:** Zhe Cui.

**Formal analysis:** Zhe Cui, Ying-Ying Tang, Myoung-Ho Lee.

**Investigation:** Zhe Cui.

**Methodology:** Zhe Cui, Myoung-Kwon Kim.

**Software:** Zhe Cui, Ying-Ying Tang, Myoung-Ho Lee.

**Writing – original draft:** Zhe Cui.

**Writing – review & editing:** Zhe Cui.

**Data curation:** Myoung-Kwon Kim.

**Project administration:** Myoung-Kwon Kim.
